# Fabrication of Multimode-Single Mode Polymer Fiber Tweezers for Single Cell Trapping and Identification with Improved Performance

**DOI:** 10.3390/s18092746

**Published:** 2018-08-21

**Authors:** Sandra M. Rodrigues, Joana S. Paiva, Rita S. R. Ribeiro, Olivier Soppera, João P. S. Cunha, Pedro A. S. Jorge

**Affiliations:** 1 INESC TEC-INESC Technology and Science, 4200-465 Porto, Portugal; up201404983@fc.up.pt (S.M.R.); jpcunha@inesctec.pt (J.P.S.C.); pedro.jorge@fc.up.pt (P.A.S.J.); 2 Physics and Astronomy Department, Faculty of Sciences, University of Porto, 4169-007 Porto, Portugal; 3 Faculty of Engineering, University of Porto, 4200-465 Porto, Portugal; 4 4Dcell and Elvesys, 75011 Paris, France; ana-rita.ribeiro@4dcell.com; 5 Institute of Material Science of Mulhouse, 68057 Mulhouse, France; olivier.soppera@uha.fr

**Keywords:** photo-polymerization, optical fiber tweezers, optical trapping, beam profile shaping, back-scattering, particle differentiation, Finite Differences Time Domain (FDTD), lorentz force, Linear Discriminant Analysis (LDA), multivariate statistical analysis

## Abstract

Optical fiber tweezers have been gaining prominence in several applications in Biology and Medicine. Due to their outstanding focusing abilities, they are able to trap and manipulate microparticles, including cells, needing any physical contact and with a low degree of invasiveness to the trapped cell. Recently, we proposed a fiber tweezer configuration based on a polymeric micro-lens on the top of a single mode fiber, obtained by a self-guided photopolymerization process. This configuration is able to both trap and identify the target through the analysis of short-term portions of the back-scattered signal. In this paper, we propose a variant of this fabrication method, capable of producing more robust fiber tips, which produce stronger trapping effects on targets by as much as two to ten fold. These novel lenses maintain the capability of distinguish the different classes of trapped particles based on the back-scattered signal. This novel fabrication method consists in the introduction of a multi mode fiber section on the tip of a single mode (SM) fiber. A detailed description of how relevant fabrication parameters such as the length of the multi mode section and the photopolymerization laser power can be tuned for different purposes (e.g., microparticles trapping only, simultaneous trapping and sensing) is also provided, based on both experimental and theoretical evidences.

## 1. Introduction

The recent developments in Biology have stressed out the need of developing devices to manipulate and differentiate micro and nano-sized particles and cells. In the search for this kind of tools, optical tweezers came along as suitable solutions, considering its ability to trap and manipulate microparticles or cells without physical contact [1,2]. Optical trapping (OT) was first reported by Arthur Ashkin in 1970 [1] when, for the first time, micro-sized particles were trapped in between two equal counter propagating beams. Later in 1986, he was able to successfully trap dielectric particles by creating a single-beam gradient force trap [3]. Since then, optical trapping has evolved and has been used in a variety of areas, starting from the manipulation of nanostructures (e.g., plasmonic particles, semiconductor nanowires, and carbon nanostructures [4]); biological-derived cells and nanometric vesicles [5]; to viruses and bacteria [2,6].

Currently, most of the widely used OT setups are based on bulky optical devices [7,8,9]. However, they are associated with several limitations as high manufacturing costs, large dimensions and low degree of portability. In this context, optical fiber tools can provide more versatile solutions towards the miniaturization of optical tweezers setups, given their small scale and precise light delivery to the source and the target. However, a specific shaping of the beam profile guided by these tools is always necessary in order to establish the adequate conditions for stable trapping effects. A diversity of microfabrication processes based on the modification of the fiber extremity according to specific patterns or the assembly of microstructures on one of its ends, have been proposed. In fact, several efforts have been made to develop dedicated microstructures able to focus the propagating laser beam onto a given spot in space at a density capable of a stable optical trapping of microparticles. These include processes such as chemical etching, thermal pulling or high resolution micromachining using Focused Ion Beam (FIB) or femtosecond laser systems [7,9]. Nevertheless, most of these methods are time consuming and extremely expensive.

Recently, in the search for a more versatile technique, we have proposed, in collaboration with Soppera et al. [10], the fabrication of spherical lenses on the top of optical fibers through a simple and low-cost photopolymerization method that enables trapping [10]. We demonstrated, for the first time, the 2D single-particle trapping of different kind of targets, including synthetic microparticles, yeasts and more complex cells (e.g., *Drosophila Melanogaster* and mammalian rodent glial neural cells), using a polymeric spherical lens fabricated through this method [7,11,12,13]. In addition to trapping, this technique also allows simultaneous trapping, manipulation and sensing of the targets. This method can then contribute towards the development of hybrid miniaturized optical devices with several applications for bioanalysis, Biology and Medicine. In this context, the light scattered by a particle is then the key to differentiate simple cells or synthetic particles, given its dependence on the target characteristics such as particle diameter, refractive index, geometry, composition, heterogeneity degree, health state of the cell, etc. With this in mind, we were able to develop, for the first time, a way to simultaneously trap a microparticle/cell and differentiate its type through the time and frequency analysis of short-term portions of the collected back-scattered signal, using the same type of polymeric tips [12,13,14]. We are currently still investigating the possible targets, for which the information provided by the acquired back-scattered signal (both in the frequency and time-domain) is reliable enough to enable differentiation of its distinct subtypes. However, in this context, it is essential that the fabricated lenses ensure a strong and stable trapping effect, since particle immobilization during a sufficient period of time is paramount for its differentiation through the collected back-scattered signal. Particle displacements due to a weak or unstable trapping can introduce noisy segments into the back-scattered signal, precluding reliable identification.

In this work, a variant of the previously reported self-guiding photopolymerization method [7,15] is presented, where a multi-mode (MM) fiber segment is introduced at the tip of the SM fiber, prior to the polymerization process, enabling a better control of the polymeric lens final features, such as thickness and curvature radius. The optimization of the lens fabrication process in order to obtain simultaneously robust trapping and reliable particle differentiation performance is addressed and a comparison study is conducted to understand how the newly developed hybrid lenses perform, by varying crucial fabrication parameters such as MM section length and photopolymerization laser power. Both theoretical and experimental evidences were collected to support our conclusions.

## 2. Methods

In this section we present and describe all the methods and equipment employed in this work. Initially, several polymeric lenses were fabricated on the top of cleaved optical fibers, having a SM-MM section, where two fabrication parameters were varied (see Section 2.1). Then, the trapping performance of the new tips on yeast cells was tested. The Drag Force method [8] was used for estimation of the trapping forces (please see Section 2.3). The differentiation ability of the type of microparticles trapped, using the back-scattered signal was evaluated for each type of micro-lenses geometry and fabrication parameters. Two classes of particles (PMMA microspheres and yeast cells) were considered to test lenses differentiation performance. All the details regarding particles type differentiation though the back-scattered signal analysis collected through fabricated micro-lenses are described in Section 2.4 and Section 2.5. Finally, and in order to understand the obtained differences according to the final geometry of the obtained lens, theoretical simulations based on the Finite Differences Time Domain (FDTD) for the propagation of the electromagnetic field were performed (please see Section 2.6).

### 2.1. Fabrication Method of Micro-Lenses by Photo-Polymerization

**Figure 1:**
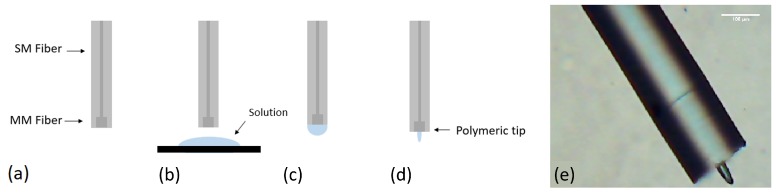
Fabrication method of the micro-structures at the end of the fiber: (**a**) it starts by cleaving two types of optical fibers: a SM and a section of a MM fiber; which are then spliced together. The resultant fiber portion is spliced once again and placed on a moving stage; (**b**) Then, it is dipped into the solution with the photo-initiator and monomer; (**c**) When the fiber is dipped out from this solution, a drop of liquid is formed on its tip; (**d**) Then, the liquid drop is exposed to the laser source, cured, and the remaining non-polymerized monomer solution portions are washed out using ethanol and the polymeric fiber tip is then formed on the top of the fiber; (**e**) Visual aspect of the final micro-lens on the top of an optical fiber.

The fabrication of microstructures at the end of the optical fiber is based on a guided wave photo-polymerization process (Figure 1) [10]. The monomer and photo-initiator used were the pentaerythritol triacrylate (PETIA) and the Irgacur 819 (associated to a working wavelength range between 375 and 450 nm), respectively.

**Figure 2:**
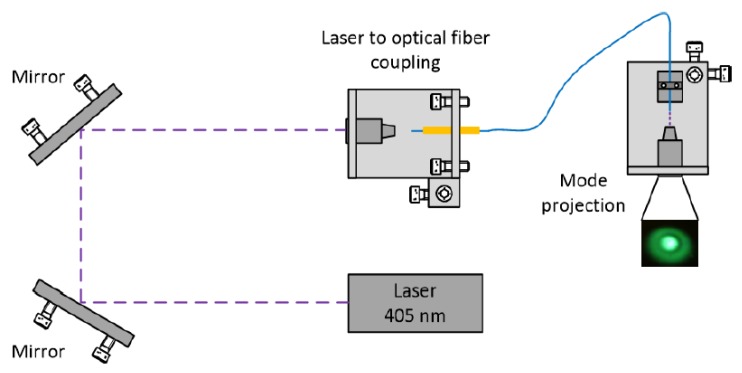
Experimental setup used in the fabrication process of the polymeric micro-structures. The optical modes were selected according to the light input angle. The light is coupled to the optical fiber using a 10X objective and a second objective is used to project the output modes onto a flat surface.

Considering this working range, a 405 nm laser diode (LuxX CW, Omicron) was used for triggering the photopolymerization. In the fabrication of the new SM+MM tip, we started by cleaving the two types of optical fiber: the SM fiber (Thorlabs SM 980-5.8-125) and a section of a MM fiber (Thorlabs MM 0.22 NA, ϕ 50 μm, 250–1200 nm). The two fiber sections were then spliced together. Afterwards, the fiber was cleaved once again, near the splice region, leaving just a few micrometers of the MM section at the tip (Figure 1a). Once this process was completed, the fiber was placed on a moving stage and dipped into the solution with the photo-initiator and monomer (step Figure 1b). A drop of liquid was then formed on its tip, which was exposed to the laser source (step Figure 1c) and cured. Then, the remaining non-polymerized monomer solution was washed out using ethanol (step Figure 1d). Since the SM 980 fiber behaves as MM at 405 nm, care had to be taken to excite a fundamental mode in a stable fashion. The optical setup used for this fabrication process is depicted in Figure 2. For laser alignment with the optical fiber, the input angle of the beam was controlled by adjusting the mirrors or the platform where the objective and the fiber stood. For this work, the LP${}_{02}$ mode was used, since it provided a more stable output with an uniform Gaussian distribution at its center.

Aiming to build a lens type micro-structure, the ideal beam shape to be imprinted on the tip, would be a gaussian one. Such uniform slow varying power distribution should result in micro-structures with lens-like shape at the tip, enabling the focus of the guided light at the fiber output at a specific point, depending on its curvature radius. In addition to the modal power distribution, other fabrication parameters can be adjusted, that influence the final shape of the polymeric lens. As previously demonstrated, the main features of the final tip depend on several microfabrication parameters such as the optical power used in the polymerization process, the exposure time [7] and, in this new particular case, the length of the multi-mode section.

**Figure 3:**
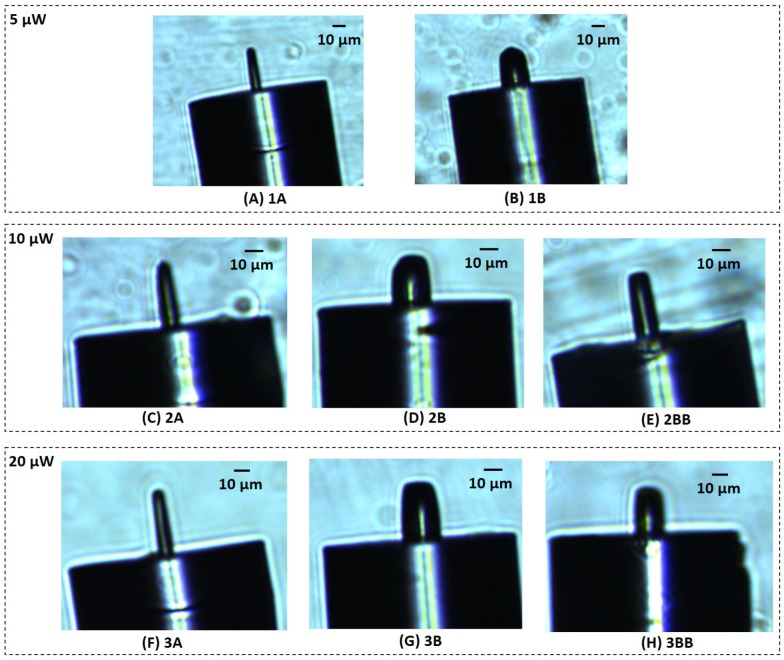
Microscopic images of the micro-lenses, obtained using SM-MM fiber tips, which were selected for detailed characterization. (**A**,**B**) Lenses fabricated using a photopolymerization laser power of 5 μW; (**C**–**E**) 10 μW and (**F**–**H**) 20 μW; (**A**,**C**,**F**) Lenses fabricated using shorter MM sections; (**B**,**D**,**E**,**G**,**H**) Lenses fabricated using longer MM sections.

**Figure 4:**
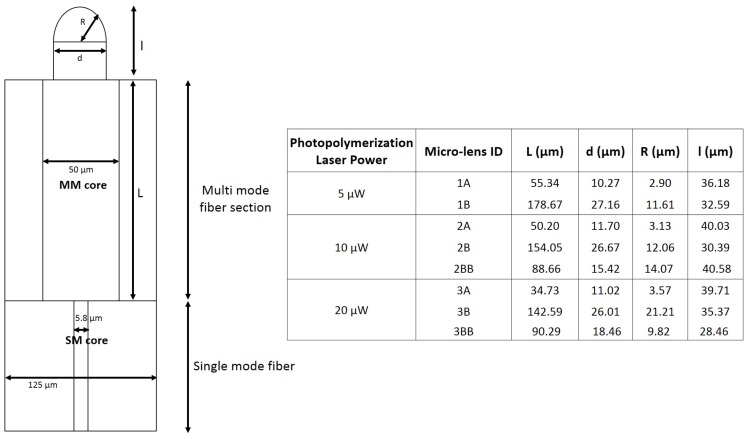
Geometrical scheme explaining the structure of the polymeric lensed fiber tips fabricated through the novel method proposed and table with corresponding dimensions. Depending on the photopolymerization laser power and the length (L) of the MM fiber section, the fiber tips will have different curvature radius R, base diameter (d) and l values.

Several optical fiber tips were fabricated using the method described above, considering three different photopolymerization laser powers: 5 μW, 10 μW and 20 μW. For each power setting, probes were fabricated having distinct lengths of MM fiber at the tip. From this fabrication options resulted a set of probes with distinct features. From all the tips fabricated, a set of 8 polymeric lensed fiber tips were selected for full characterization. Selection was made, considering for each polymerization laser power, probes with distinct features, representative of the different sub-types obtained (according to thickness, curvature radius, etc.). In particular, the selected probes were characterized in terms of beam propagation properties through theoretical simulations, trapping performance and particle differentiation ability using the back-scattered signal. The selected subset is detailed in Table 1, where it can also be seen the type of analysis performed with each micro-lens type. Their visual aspect is presented in Figure 3. The geometrical parameters characterizing each probe (e.g., micro-lens curvature radius) were obtained from microscopic images using ImageJ^®^ software, version 1.51j (please see Figure 4).

### 2.2. Optical Trapping and Back-Scattered Signal Acquisition Setup

**Figure 5:**
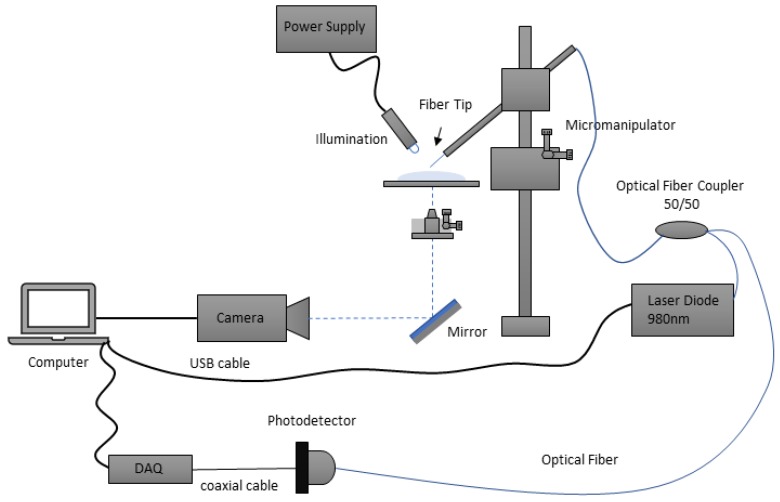
Experimental setup used in the trapping and back-scattering signal acquisition experiments.

The experimental setup used to trap and manipulate the particles under test is depicted in Figure 5. It consists in an inverted homemade microscope with a 20× objective, connected by a mirror to a CMOS camera. The fiber that contains the micro-lens is controlled using a motorized micro-manipulator capable of handling the optical fiber in the x, y and z direction, and varying the inclination angle. Since the fiber is too fragile to be handled directly by the micro-manipulator, it was first inserted in a capillary, and only then placed on the controller. Trapping can only occur at tilt angles above 30${}^{\circ }$, therefore the capillary holding the fiber was tilted to approximately 55${}^{\circ }$, to ensure stable trapping [7]. The lensed fiber was connected through the exit port of a 1 × 2 (50:50) optical coupler to a pigtail 980 nm laser diode (500 mW, Lumics, Berlin, Germany) and the signal acquisition module. The latter included a photodetector to collect the back-scattered signal, and a data acquisition board (DAQ) from National Instruments for signal processing. A computer controlled both the DAQ and the laser enabling control of its input and output parameters. This configuration allowed both laser light guidance through the optical fiber, and the acquisition of the back-scattered signal through the photodetector. The output laser power was set to ≈15 mW during the trapping experiments, to avoid damaging of biological cells (yeasts), but yet ensuring a stable trapping. The laser diode 980 nm signal was modulated by a sinusoidal signal with a fundamental frequency of 1 kHz, which was injected in the laser module using the DAQ.

After the above setup was correctly mounted, a simple assay was conducted for microparticles manipulation and (in some cases) back-scattered signal acquisition. A drop of one of the two solutions prepared for this experiment (distilled water containing 8 μm PMMA microparticles or distilled water with yeast cells in suspension) was placed over a glass coverslip that was then placed over the inverted microscope-based setup described above. Afterwards, each one of the fabricated micro-lenses was dipped into the solution and the laser was turned on for particles trapping and manipulation.

### 2.3. Optical Trapping Forces Experimental Calculation

Trapping forces exerted by the fabricated micro-lenses on yeast cells were determined through an adapted version of the Drag Force method, based on the Faxen’s Law, a revised version of the Stokes equation [8]. This novel version, differently from the original one, takes into account the proximity of the trapped particle to a boundary (in this particular case, the glass slab) [16]. Thus, the trapping force exerted by a microparticle can be described by the sum of the inertial and drag forces:

(1) $$F_{T}=F_{inertial}+F_{drag}=m\frac{\partial^{2}s}{\partial t^{2}}+6\pi \xi \eta r\frac{\partial s}{\partial t},$$

in which *m* is the mass of the trapped particle, s(t) represents the particles trajectory during manipulation, ξ a correction factor due to the proximity to the trapping chamber (in this particular case, ξ=3.08) [8,16]), η the viscosity of the media (in this case, distilled water, $8.90\times 10^{-4}$ Pa) and *r* is the radius of the particle. However, the inertial force can be considered negligible, taking into account the low Reynolds number associated with this situation ($Re\approx 10^{-59}$). Thus, for a particle in equilibrium (stably trapping) there must be a balance between the trapping and the drag forces. We can therefore calculate the total trapping force exerted on the particle by calculating the drag force [8,16]:

(2) $$F_{T}=6\pi \xi \eta r\frac{\partial s}{\partial t}.$$

Once the viscosity of the media η, the radius of the particle *r* and the correction factor ξ are known variables, it is only needed to calculate the velocity of the particle ($\frac{\partial s}{\partial t}$) when manipulated, to calculate the corresponding trapping forces.

**Figure 6:**
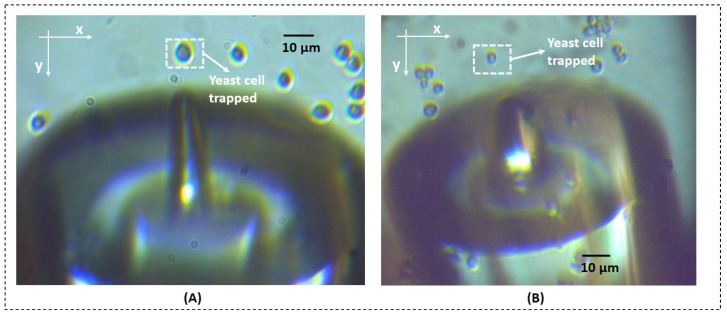
Yeast cells stably trapped using the fabricated micro-lenses (**A**) 2A and (**B**) 3BB.

In order to experimentally calculate trapping forces exerted by each one of the fabricated micro-lenses, the following sequence of events was recorded by the CMOS camera at a sampling rate of 13 frames per second. Since our polymeric micro-lenses are only able to trap microparticles in two dimensions [7,13,16], the gradient force is the trapping force component which plays the major role in the trapping phenomena. Considering our previous studies involving theoretical simulations [13,17,18], the longitudinal component relatively to the optical axis of the gradient force is significantly weaker in comparison to the transversal one. For this reason, only transversal displacements of the particles/yeast cells due to optical trapping (towards the left and right directions in relation to the optical axis, −x and +x directions, respectively) were analyzed and transversal gradient trapping forces calculated and compared between micro-lenses with different geometries. Once a yeast cell was stably trapped as depicted in Figure 6A,B, the laser was turned off and the polymeric tip was displaced towards the left (−x) or right (+x) by a few micrometers away from the cell. Then, the laser was turned on and the yeast was attracted back to the trapping equilibrium position in front of the displaced tip. This was repeated, in average, for three times for each direction (towards −x and +x) while yeast cell movements were recorded using the CMOS camera, controlled by the IDS^®^ *uEye Cockpit* software, at a sampling rate of 13 frames per second. Trapping forces characterization was performed considering a laser power of ≈15 mW and a fiber tilt angle of ≈55${}^{\circ }$.

After particles tracking, it was possible to obtain the yeast cells positions along time, by importing the acquired frames to Matlab R2015a^®^ using the dedicated software *CellTracker* [19]. The particles position along time, corresponding to the restoring movement due to the particles attraction towards the trapping equilibrium point, was then fit to the Langevin equation, to obtain smoother curves [7,13,20]. Then, trapping forces profile for yeast cell displacements towards −x and +x direction were obtained by calculating the restoring movement velocity through the Equation (2). The profile of transversal trapping forces exerted on yeast cells in distilled water were compared between the polymeric tips 1A, 2A, 3A, 1B, 2B and 3B.

### 2.4. Back-Scattered Signal Acquisition and Processing

**Figure 7:**
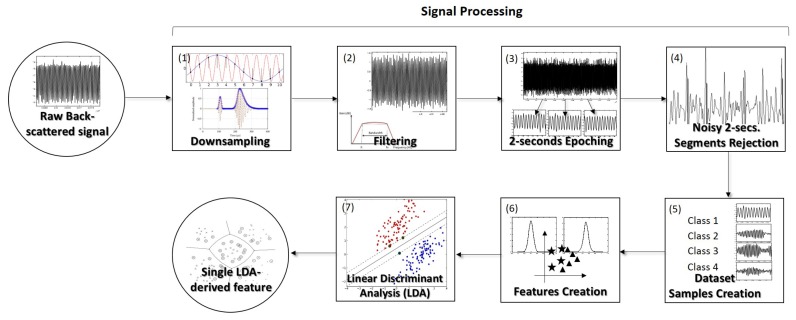
Scheme explaining back-scattered signal processing and post-processing steps. (**1**) After the acquisition of 60-s of back-scattered signal for each particle (PMMA and yeast cells), the acquired signals were downsampled from 100 kHz to 5 kHz; (**2**) Then, each whole acquisition was filtered using a 500 Hz high-pass Butterworth filter; (**3**) The 60-s segments of signal were divided into short-term portions of 2 s; (**4**) Noisy 2-s short-term signal portions were therefore removed taking into account the *z-score* value calculated before epoching. Portions whose value(s) exceeded |z-score| > 10 were removed at this stage; (**5**) After signal processing steps, our dataset was composed by 2-s short-term portions of back-scattered signal; (**6**) A set of more than 40 time- and frequency-domain features characterizing each 2-s portion was therefore created and assigned to each particle class; (**7**) Then, the Linear Discriminant Analysis (LDA) was applied to gather all the relevant information provided from the original set of features in a single one, in order to facilitate interpretation by an interrogation sensing system. At the end, the proposed system would be able to provide the values corresponding to that single-feature, resulting from a multivariate combination of the parameters calculated in (**7**).

In order to investigate the particles differentiation ability of each type of lens using the back-scattered signal, a simple assay was performed for six of the 8 fabricated micro-lenses: 1A, 1B, 2A, 2BB, 3A and 3BB. After frames acquisition for calculation of the trapping forces, a new drop of solution (from the two prepared for the differentiation problem: 8 μm PMMA microparticles in distilled water or yeast cells in distilled water) was placed over a new glass coverslip. Then, each micro-lens was again dipped into the solution and, once the particle (yeast or PMMA microsphere) was stably immobilized in front of the fiber tip due to optical trapping, 60 s of back-scattered signal were acquired at a sampling rate of 100 kHz, using the configuration depicted in Figure 5. This was performed for each particle class and each one of the 5 cells/microparticles of each class used (5 PMMA microspheres and 5 yeast cells). After signal acquisition, a sequence of signal processing steps was applied to each 60-s whole acquisition, considering a signal processing pipeline already applied in previous studies successfully conducted by our lab [12], using single mode fiber tweezers. These steps are summarized in the scheme of Figure 7. Custom-built scripts from Matlab^®^ involving functions from Signal Processing and Statistics toolboxes were developed for both signal acquisition and processing.

After signal acquisition, and due to the high computational complexity inherent to the high sampling rate of the original signal (100 kHz), each 60-s acquisition was downsampled to 5 kHz. Then, once the input laser signal was modulated by a 1 kHz sinusoidal modulation signal, a second order Butterworth high-pass filter with a cutoff frequency of 500 Hz was applied to each acquisition, to remove low-frequency noise components such as the 50 Hz electrical grid-derived component. Then, in order to generate a robust parameter which could be able to correctly differentiate the type of particle trapped as fast as possible, each entire 60-s acquisition was split into short-term portions of 2 s. In order to remove noisy 2-s signal portions from the post-processing analysis, the 2-s segments whose z-score values (calculated before epoching and therefore considering the whole 60-s acquisition where each segment belonged to) exceeded the threshold |z-score| > 10 were not included in the post-processing stage. In this way, a dataset containing several 2-s short-term back-scattered signal portions for each one of the two classes considered—PMMA and yeast cell trapped - was obtained after signal acquisition and processing according to the steps 1–4 from Figure 7. Then, a set of 43 time- and frequency-domain features characterizing each 2-s short-term signal belonging to each class was created—please see Section 2.5.1 for more details about the extracted parameters. Considering that it could be a hard task for a sensor reading system to directly aggregate all the relevant information provided from the 43 distinct parameters and once a linear combination from those attributes has already revealed to be suitable for particles type differentiation [12,14], we applied the Linear Discriminant Analysis (LDA) to gather the relevant information into a single parameter. This method is therefore able to generate a single feature that results from a linear combination of a set of original features, in way that better separates data classes. Afterwards, the particles type differentiation ability considering this single feature was compared between the six micro-lenses described in Table 1.

### 2.5. Microparticles Type Differentiation through Linear Discriminant Analysis (LDA)

#### 2.5.1. Extracted Parameters/Features

As mentioned above, a set of 43 features based on the time- and frequency-domain information contained in each 2 s signal portion was extracted from each signal segment. This group of features was carefully selected to be part of the LDA method, and have already showed to be relevant for particles class differentiation in previous studies also conducted by our lab [12,14]. The extracted features can be divided into two main types: time- and frequency-domain. Considering each main type, they can also be divided into two subtypes. Time-domain characteristics were therefore divided into time-domain statistics attributes and time-domain derived parameters (please see Table 2). In terms of frequency domain analysis, considering that the widely used Fast Fourier Transform (FFT) could introduce discontinuities in the original signal to generate the corresponding spectrum [21] due to its short duration, the Discrete Cosine Transform (DCT) [22] and Wavelet functions [23] were used instead.

The time-domain statistics features used to characterize each 2-s signal portions are summarized in Table 2. Considering that the *Nakagami* distribution is considered a suitable approximation to the time-domain histogram of light scattering phenomena in Biology [24], the Probability Density Function (PDF)-derived $\mu_{Nakagami}$ attribute that better fits each 2-s signal portion histogram to the Nakagami distribution was also used as a feature to characterize each signal segment.

In terms of the frequency domain, and considering that the DCT is able to embed most of the signal energy into a small number of coefficients, it was possible to obtain the first *n* coefficients by computing [22]:

(3) $$E_{i}^{DCT}\left[l\right]=\sum_{k=0}^{N-1}\varepsilon_{i}\left[k\right]\cos\left[\frac{\pi l(2k+1)}{2N}\right],\;for\;l=1,\ldots ,n\;,$$

in which $\varepsilon_{i}$ is the signal envelope estimated using the Hilbert transform. The percentage of the total amount of the signal energy which each coefficients set represents (from the most to the *n*th representative one) can be determined by sorting the DCT coefficients from the highest to the lowest value of magnitude and obtaining the following vector [22]:

(4) $$y_{i}={\left(E_{i}^{DCT},\ldots ,E_{i}^{DCT}\left[l^{n}\right]\right)}^{T}\;,$$

in which $E_{i}^{DCT}\left[l^{1}\right]$ represents the highest DCT coefficient in magnitude. The percentage of the total amount from the original signal energy represented by the first till the *n*th coefficient of the vector (4) can be obtained by dividing the norm of the vector formed by the first till the *n*th coefficient by the norm of the vector comprising all the coefficients. Based on this definition, the following DCT-derived parameters were extracted from each 2-s signal portion: the number of coefficients needed to represent about 98% of the total energy of the original signal ($N_{DCT}$), the first 20 DCT coefficients extracted from the vector defined in (4), the Area Under the Curve (AUC) of the DCT spectrum from 0 to 2.5 kHz ($AUC_{DCT}$), the maximum amplitude of the DCT spectrum ($Peak_{DCT}$) and the DCT-derived power spectrum within the frequency range analyzed (from 0 to to 2.5 kHz) ($P_{DCT}$).

The remaining parameters used for particles differentiation were based on 2-s short-time signal portions frequency decomposition using wavelets [25]. Signal decomposition using wavelets is a broadly used signal processing method since allows frequency subband decomposition with the possibility to retain relevant temporal-spectral properties from the original signal [25]. Two mother Wavelets—*Haar* and Daubechies (*Db10*)—were selected to characterize the back-scattered signal portions, taking into account their simplicity and since they were already successfully used to decompose back-scattered signals in underwater scenarios [22,26]. Six features for each type of mother Wavelet based on the relative power of the Wavelet packet-derived reconstructed signal (one to six levels) were therefore extracted from each 2-s back-scattered signal portion, totaling 12 features (Table 2) [22,26].

#### 2.5.2. The Linear Discriminant Analsyis (LDA): Towards a Single Parameter for Particles Differentiation

Considering that evaluating all the 43 parameters extracted above would be a hard task for a sensing reading system, we decided to apply a multivariate statistical method, usually used for features dimensionality reduction. This method allowed gathering all the relevant information contained in the 43 features into a single parameter [27,28]. This single parameter resulted from a weighted linear combination of all the original 43 parameters [27]. The weight given to each original variable depended on its relevance for the trapped microparticles type differentiation problem. In this case, we were interested in the best linear combination between the 43 features which better separated the two classes involved in the problem: “yeast cell trapped” or “PMMA microsphere trapped”. Thus, considering the *N*-dimensional features space (in which *N* represents the number of original features, N=43), the LDA tries to find the projection hyperplane that minimizes the interclass variance and maximizes the distance between the projected features means of the classes [27,28,29]. With the LDA, we were able to find a subspace of lower dimension (in this particular case, a single dimension), in which the data points of the original problem are “separable”. However, due to intrinsic amplitude differences between features and in order to project them to the same values space range, a normalization procedure was applied to each sample of the dataset before the LDA. The samples mean value across each feature was subtracted to each data sample from that feature, and then divided by the corresponding feature standard deviation [28]. The Statistics Toolbox from Matlab 2015a^®^ was used to perform the LDA.

#### 2.5.3. Statistical Analysis

In order to evaluate the differentiation ability of the novel LDA-based single parameter, a Student T-test was performed to verify if there was a significant difference between the two classes considered for the novel feature. This statistical test was performed for all the micro-lenses for which the back-scattered signal was acquired (lenses 1A, 1B, 2A, 2BB, 3A, 3BB). The statistical significance level of 0.05 was considered for all the tests performed [30]. The obtained *p*-values regarding the parwise comparison were compared between the micro-lenses. The smaller the *p*-values, the higher it was the classes differentiation ability of each micro-lens considered [30].

### 2.6. Computational Model and Theoretical Simulations

To better understand the effect of the inclusion of a multi-mode section in the polymeric fiber tweezers as well as the several different photopolymerization laser power values, we also performed theoretical simulations of the propagation of the trapping beam along the fabricated tweezers. Simulation parameters (computational grid, waveguide geometry, laser source) were set to match, as closer as possible, the real experimental conditions. The stationary electromagnetic field was propagated (in two dimensions) along six different micro-lenses: 1A and 1B (for 5 μW microfabrication laser power); 2A and 2B (10 μW); and 3A and 3B (15 μW), considering a simulation environment composed by a 500 × 70 μm computational grid (please see Table 3, where all the simulation parameter values considered are listed).

**Figure 8:**
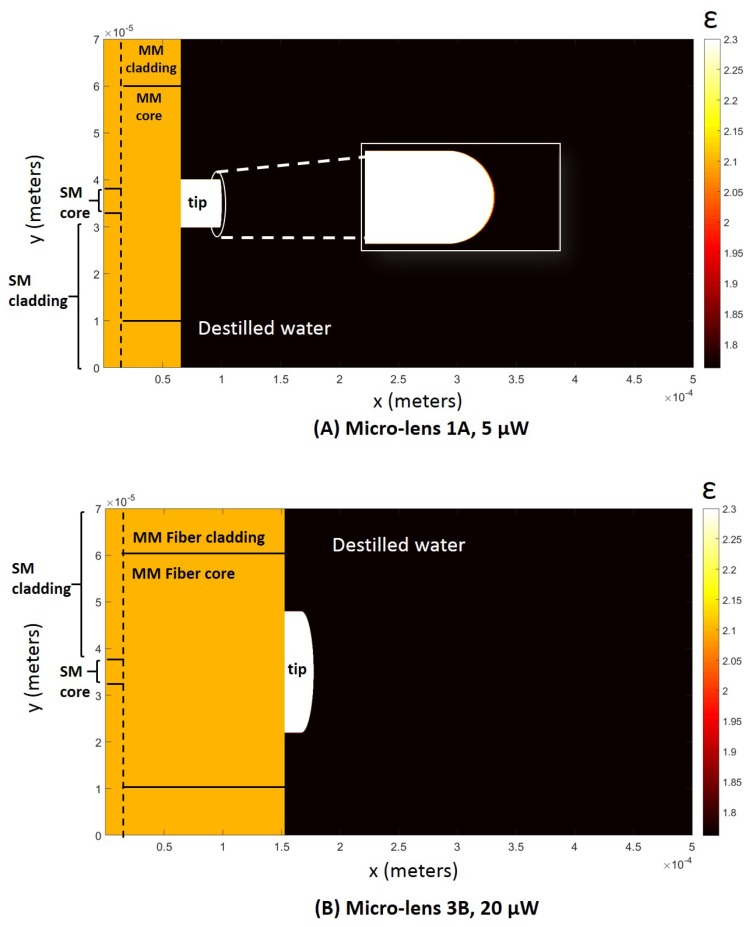
Representation of the simulation environment for (**A**) 1A fiber tip; (**B**) 3B fiber tip.

Field propagation considering each one of these micro-lenses was performed using the Finite Differences Time Domain (FDTD) method implemented on the MEEP software [31] compatible with Python^TM^. The electromagnetic field was propagated long enough to obtain a stable solution considering the designed computational grid. In Figure 8 it is possible to observe a representation of the simulation environment considered.

## 3. Results and Discussion

### 3.1. Characterization of the Polymeric Lensed Fiber Tweezers with Multi-Mode Section

The main goal of this work was to enable a better control of the tip fabrication process, namely increasing its robustness and ability to control the curvature radius and thickness. In this framework, several micro-lenses were produced considering different lengths of multi-mode section at the tip (from 20 μm to 180 μm). This was done for three distinct photopolymerization laser powers: 5, 10 and 20 μW (values measured at the output of the fiber, before polymerization). All other microfabrication parameters were maintained at a predetermined value: concentration of photo-initiator in the solution of ≈0.2%, exposure time of ≈10 s and excitation of the LP${}_{02}$ mode.

Since the idea is to improve the robustness and quality of the resulting micro-structures, it is crucial to understand how the multi-mode section can affect the resulting micro-lens geometry. Assuming a diffraction limited laser beam, and the different core dimensions of the optical fibers (SM980 of 5.8 μm and MM of 50 μm), it is expected an expansion of the optical beam when propagating from the SM fiber to the MM segment.

**Figure 9:**
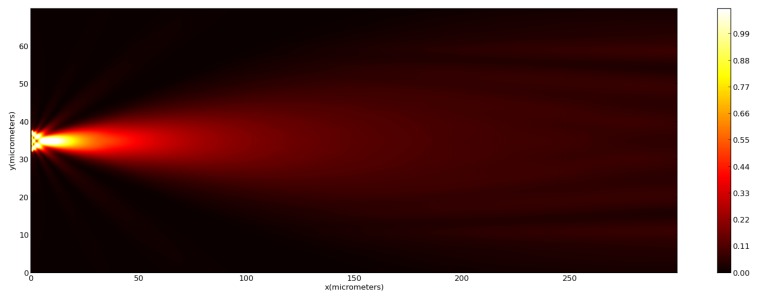
Propagation of the photopolymerization laser beam on the MM optical fiber core at 405 nm (photopolymerization wavelength).

In Figure 9 we present a simulation of the laser beam intensity distribution inside the multi-mode optical fiber core during the photopolymerization fabrication process (@405 nm). It is possible to observe the initial spread of the beam through the core until filling in the entire core diameter. The resulting beam expansion can then be calculated through the numerical aperture of the optical fiber, considering the following two equations:

(5) $$N.A.=n_{1}\sin\theta_{m}=\sqrt[{}]{n_{co}^{2}-n_{cl}^{2}},$$

(6) $$d_{s}=2L\sin\theta_{m}+5.8,$$

and through which is possible to predict how much the initial beam will spread given a certain MM length *L*, and thus determine the diameter ($d_{s}$ in μm) of the laser spot at the tip output. $n_{1}$, $n_{co}$ and $n_{cl}$ are the refractive indexes of the SM fiber core, MM fiber core and MM fiber cladding, respectively; $\theta_{m}$ represents the maximum acceptance angle of the fiber; and the value of 5.8 μm corresponds to the SM fiber core radius. Considering the specifications of the optical fiber, $\theta_{m}=7.59^{\circ }$. The light spot diameter at the fiber output will consequently determine the dimension of the micro-structure base, enabling to control its thickness and robustness.

**Figure 10:**
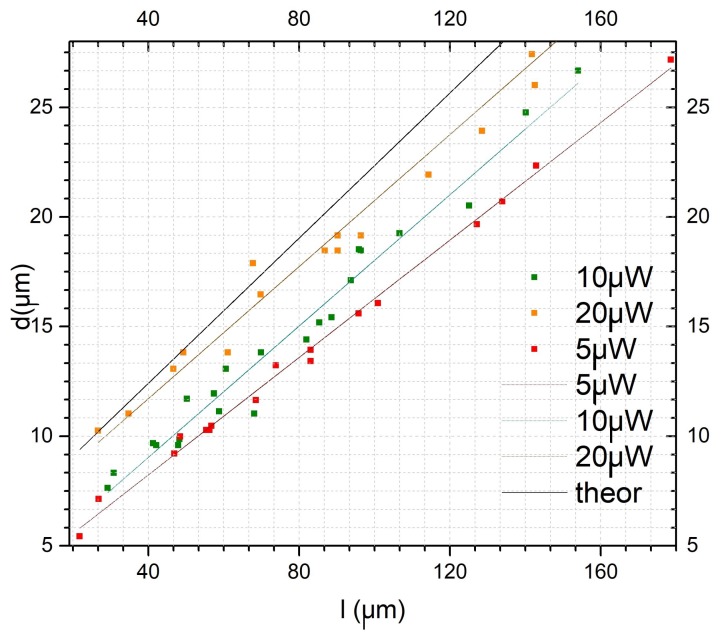
Micro-lens diameter evolution as a function of the MM section length. *theor*—theoretical line.

The several tips fabricated with variable power and MM section length were fully characterized. The graph presented in Figure 10 shows the experimental results concerning the geometrical properties of the tips obtained, together with a theoretical curve derived from Equations (5) and (6). Namely, it is represented by the diameter of the polymeric tip *d*, at its base, as a function of the length of the MM section used. It can be seen a general agreement in the linear tendency between MM length and corresponding diameter. However, there is a slight offset and some mismatch in the slope of the experimental curves. Nevertheless, a closer observation also shows that the experimental values converge to the theoretical curve as the polymerization power increases. This convergence is also confirmed by the fitting parameters obtained for each values set (Table 4). With increasing power, an increase in the slope of the experimental fit was also verified, in a way that it approaches the theoretical expected value of 0.165 (please see Table 4). The same convergence behavior is observed for the fitting offset. Another interesting result is related to how the difference between theoretical and experimental values for the base diameter expands as the polymerization power increases, growing from 3.83 μm considering a MM section of 48.42 μm, to 8.20 μm for a MM section of 178.67 μm. A possible explanation is related to the Gaussian behavior of the laser beam. As observed through the theoretical simulation of the photopolymerization beam on the MM section (Figure 9), the beam spreads as it propagates through the section, which is also translated into the damping of the Gaussian profile maximum peak magnitude. This reduces the beam effective polymerization section, making the experimental values not fitting with the theoretical case. It is because, for simplicity, it was considered that the full Gaussian beam radius was capable of polymerizing. In this context, we would expect that a higher polymerization power would led to the fabrication of micro-lens with more suitable features for both beam shaping and trapping, due to closer affinity between theoretical and experimental parameters. However, the role of the optical power plays a dual influence in the polymeric tip final properties. Indeed, another parameter that must be considered to analyze micro-lenses performance is the curvature radius of the tip. Since the desired structure must have suitable lens-like properties, it is desirable to obtain curved, spherical-like tips, suitable to adequately focus the beam onto a circular spot. In this context, the selection of the optical fiber mode to excite during photopolymerization must be a tightly controlled process. All the tips considered along this study were fabricated using the LP${}_{02}$ mode, which had a Gaussian shape at the center followed by two bilateral and symmetrical minimums on both sides. This was done because it was observed that the excitation of this mode was much more stable than fundamental mode excitation, which was easily contaminated by higher order contributions. The described conditions should result in the fabrication of micro-lenses with suitable characteristics, however other factors also influence the features of this micro-structure, beyond the photopolymerization laser power, such as the ratio between the photoinitiator and monomer concentrations. In the end, the resulting tip curvature radius, is controlled by a combination of factors. In this context, even with relatively stable fabrication conditions, it was observed some degree of variability in the resulting tip curvature radius.

**Figure 11:**
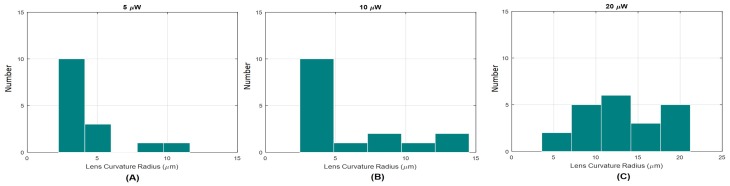
Histograms representing the distribution of final micro-lenses radius considering photopolymerization power values: (**A**) 5 μW; (**B**) 10 μW and (**C**) 20 μW.

The distribution of the produced micro-lenses, according to each fabrication optical power is depicted in Figure 11, representing all the tips characterized in Figure 10 and thus including a full range of possible MM-section lengths for each power. The obtained histograms showed that, for lower polymerization power values, the curvature radius of the produced tip falls more often beneath the 10 μm range. Additionally, it is possible to conclude that the higher the photopolymerization laser power, the lower the probability of producing micro-lenses with smaller radius, giving place to spherical tips with larger radius. Considering this characteristic behavior, the tips obtained through the proposed fabrication method were categorized into two different types: Type A: lenses with base diameters lower than 15 μm—such as, for example, lenses 1A, 2A and 3A (please see Table 1)- and type B: lenses with base diameters higher than 15 μm—such as, for example, lenses 1B, 2B, 2BB, 3B and 3BB (please see Table 1). The performance of both types of lenses (Types A and B) in terms of optical trapping and particles types differentiation ability were therefore evaluated.

### 3.2. Optical Trapping Forces

**Figure 12:**
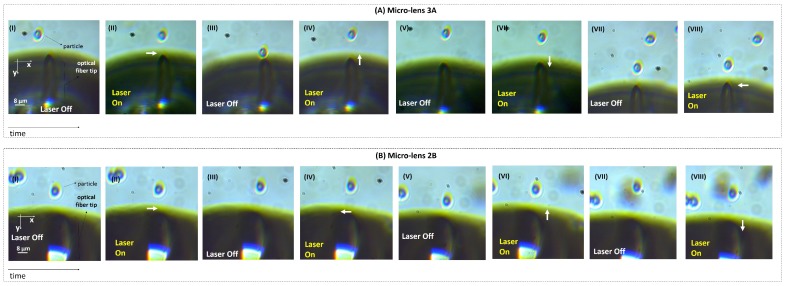
Snapshots showing the stable trapping of yeast cells at an optical power of ≈15 mW, @980 nm by micro-lenses (**A**) 3A and (**B**) 2B, fabricated using photopolymerization laser power values of 10 μW and 20 μW, respectively. (A-I,II) After the laser is turned on and the micro-lens displaced to the right, the yeast cell moved towards the right (+x direction) due to optical trapping. (A-III,IV) Then, the yeast cell is displaced towards the −y; (A-IV,V) +y and (A-VI,VIII) −x directions, by performing a similar assay. (B, I-VIII) Snapshots showing yeast cell displacements towards the +x, −x, −y and +y directions, due to trapping forces exerted by the lens 2B, respectively.

**Figure 13:**
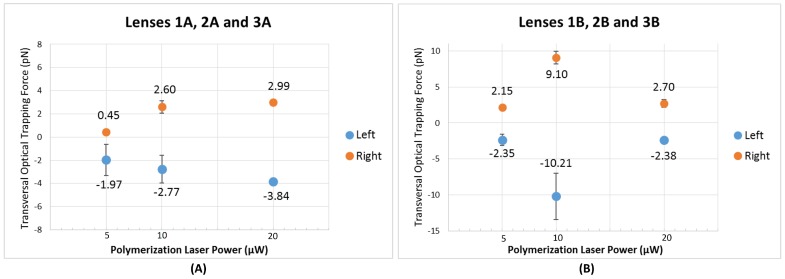
Maximum transversal trapping forces (at an optical power of ≈15 mW, @980 nm) for (**A**) lenses with smaller base diameters (1A, 2A and 3A); and (**B**) lenses with larger base diameters (1B, 2B and 3B), when the particle is moved towards the −x (left) and +x (right) directions due to optical trapping, according to the polymerization laser power value used in its fabrication.

The optical trapping ability of selected tips was also evaluated (1A, 2A, 3A, 3B, 3A and 3B). It was verified that all the produced micro-lenses ensured a stable trapping of both types of particles considered (8 μm PMMA microspheres and yeast cells). The optical trapping performance was evaluated for both transversal and longitudinal directions in relation to the optical axis, considering all the fabricated tips. However, and as already mentioned above, only transversal trapping force profiles were compared between the different micro-lenses. Video snapshots showing the stable trapping of a yeast cell (at an optical power of ≈15 mW, @980 nm) considering both directions for lenses 3A and 2B (fabricated using laser power values of 20 μW and 10 μW, respectively) are depicted in Figure 12, as examples. A graphical representation of the maximum optical force exerted on the yeast cell by each micro-lens for target displacements towards the left (−x direction) and the right (+x direction) is provided in Figure 13.

**Figure 14:**
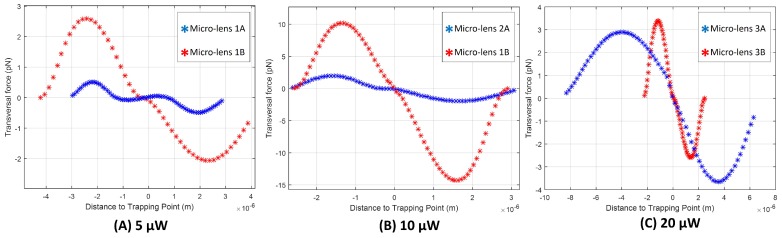
Transversal trapping forces profile (at an optical power of ≈15 mW, @980 nm) according to the distance to the trapping point for the micro-lenses fabricated considering a photopolymerization laser power of (**A**) 5 μW; (**B**) 10 μW and (**C**) 20 μW.

For Type A micro-lenses (Figure 13A), which result from shorter MM lengths, and have a thinner base, it can be observed that as the photopolymerization power values are increased, a slight improvement in the maximal magnitude value of the optical force exerted on yeast cells considering both displacement directions, is observed. Looking to Type B micro-lenses, however, which result from longer MM section, and have thicker base, there is no clear dependence with the optical polymerization power. Optical trapping force increased from 5 μW to 10 μW, but decreased from 10 μW to 20 μW, to values similar to the ones obtained with tip type A (for fabrication laser power of 20 μW). Trapping forces profile for each lens can be found in Figure 14.

Nevertheless, the best absolute performance in terms of trapping ability was achieved by the lens 2B with a larger base diameter and fabricated using a photopolymerization laser power of 10 μW. It ensured a mean maximal force 75% higher in relation to the average maximal force value exerted by the lenses type B and the other type A lenses fabricated with different laser powers. Lens 2B exerted an average maximal force of 9.10 ± 1.52 pN and −10.21 ± 5.55 pN for displacing the yeast cell towards the right and left, respectively.

Looking at the force profiles of Figure 14, it can be observed that lenses type B are predominantly more suitable for trapping (exerting trapping forces with higher magnitude values) than type A. The only exception happens for the photopolymerization laser value of 20 μW. For these conditions, lenses 3A and 3B exerted a similar trapping force in magnitude on the yeast cell.

Using a computational model proposed in previous studies conducted also by our lab, based on the Barnett and Loudon method [7,13,16,17,18,32], a representation of the field output profiles of the different types of tips fabricated was calculated.

**Figure 15:**
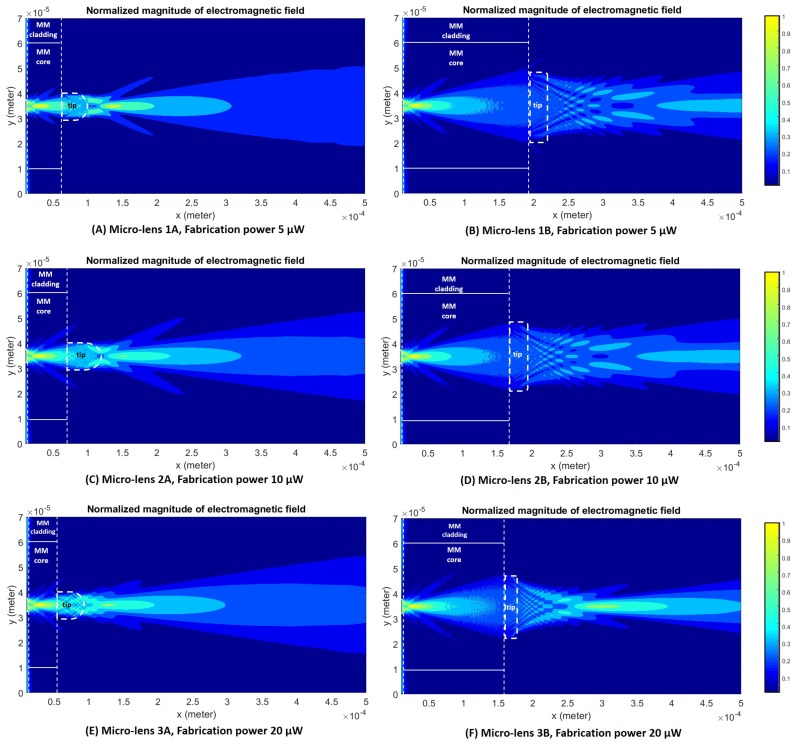
Propagation of the electromagnetic field considering the several examples of fabricated micro-lenses (1A, 1B, 2A, 2B, 3A and 3B), considering different MM section length values and photopolymerization laser powers.

The 2D graphical representations of the electromagnetic field propagation considering each one of the lenses evaluated regarding the optical trapping stability and performance are depicted in Figure 15. As expected, factors such as micro-lens curvature radius or MM section length influenced the location and distribution geometry of the beam focus spot. Clearly, there is a strong difference between the distribution of the field intensity after the beam being focused using a lens type A and type B. Lenses type A led to the focus of the input laser beam onto a highly concentrated spot in terms of energy per spatial unit, closer to the tip. Lenses type B, on the other hand, having a larger tip curvature radius, have less focusing power, resulting in focal points farther away from the tip. This way, these probes show a more disperse and heterogeneous electromagnetic field pattern in space closer to the tip, less so for the case of lens 3B. For lens 3B, the electromagnetic field propagation distribution was very similar to a lens type A, being characterized by a stronger focus. This happens probably because of its shorter length in comparison with the other lens type B, in spite of its higher curvature radius (see Figure 4). Thus, lens 3B has a more concentrated field distribution at its input, resulting in a comparatively more concentrated output.

Once the electromagnetic field was propagated for the selected tips, theoretical considerations about how they would possibly differ in terms of trapping ability were made. The Barnett and Loudon method [32] considers that the optical force exerted on each particle can be modeled as a Lorentz force exerted on the microscopic dipoles which compose the particle, being dependent on the integral of the electromagnetic field gradient over the particle. Thus, the higher the value of the electromagnetic field gradient, the higher the force exerted on the particle. However, on contrary of the three lenses type A and lens 3B, lenses 1B and 2B, having a lower focusing power, show a more spread field pattern, showing a more disperse electromagnetic field distribution in the vicinity of the tip, suggesting a higher number of possible trapping points (transversal zones of consecutive electromagnetic field maximum and minimum values and, thus, high gradient values), than for the other tips. In fact, Figure 15B,D suggest several possible transversal cuts corresponding to apparent trapping points, along which the electromagnetic field gradient can be compared between tips. Since this is a multiple solution problem, no objective conclusions could be found relatively to the theoretical trapping performance of these novel tips, neither it could be confirmed if the proposed theoretical model fits with the experimental trapping forces.

Globally, in spite of some variability in the fabrication process, the new method of polymeric tips fabrication, where a MM segment is introduced on the SM fiber tip, led to more robust micro-lenses able to exert trapping forces with magnitudes corresponding to higher values improved from twice (type A, 10 and 20 μW photopolymerization laser power) to ten fold (type B, 10 μW laser power) compared with the forces exerted by equivalent tips fabricated on simple SM fibers (conventional fabrication method) [7,16,20].

### 3.3. Trapped Microparticles Differentiation Ability Using the Back-Scattered Signal

The different types of microlenses fabricated were also tested for their ability to collect the back-scattered signal, from the trapped particles, and based on the analysis of this signal differentiate the type of microparticles. Due to practical limitations, in some cases, new lenses, with similar properties, were used in these tests (2BB and 3BB).

**Figure 16:**
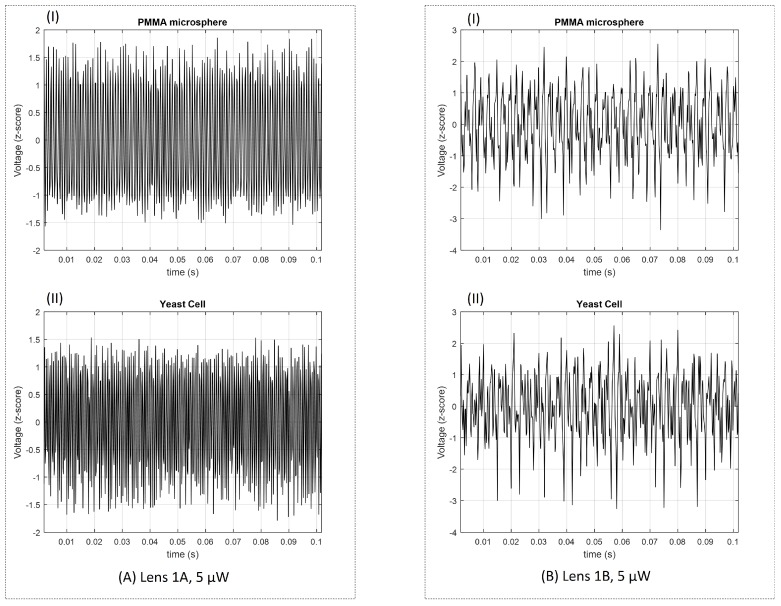
Sketches of the back-scattered signal acquired for (I) PMMA microsphere and (II) yeast cell for (**A**) micro-lens 1A and (**B**) 1B (fabricated using a polymerization laser power of 5 μm).

**Figure 17:**
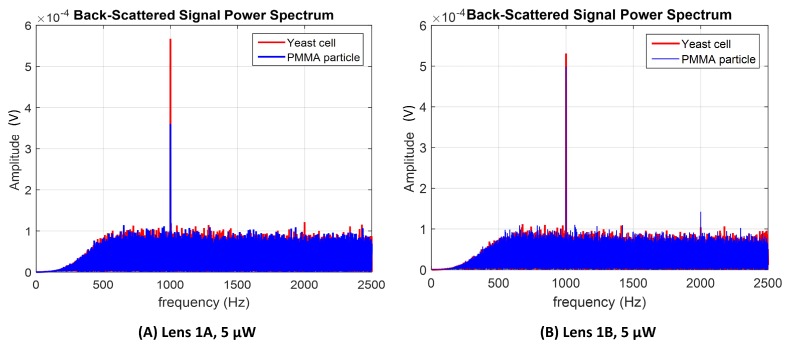
Power spectrum of filtered back-scattered signal portions of 60 s acquired using (**A**) micro-lens 1A and (**B**) 1B (fabricated using a polymerization laser power of 5 μm).

**Figure 18:**
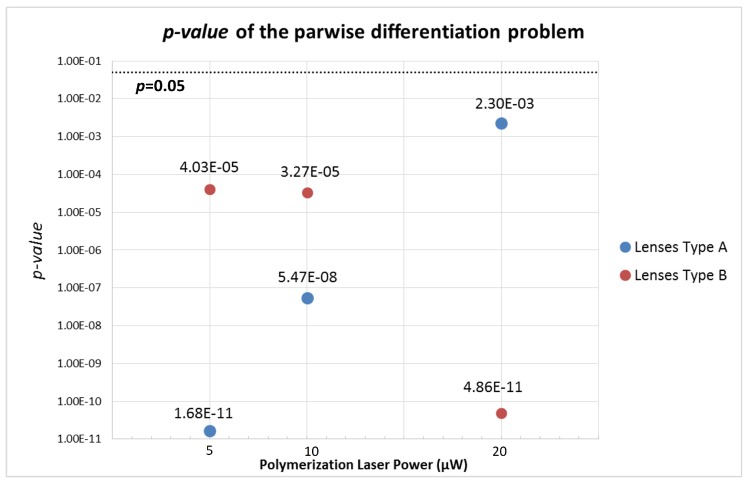
*p*-value of the statistical parwise comparison (PMMA microspheres versus yeast cells) for lenses Type A (blue dots) fabricated using laser power values of 5, 10 and 20 μW (lenses 1A, 2A and 3A, respectively) and Type B (red dots) for photopolymerization laser power values of 5, 10 and 20 μW (lenses 1B, 2BB and 3BB, respectively). Significance level: p=0.05.

Sketches of the back-scattered signal portions acquired with the different type A and type B lenses (for 5 μW photopolymerization laser power) are depicted in Figure 16. Sketches for lenses type A and B for the other polymerization laser values are provided in Appendix A. The power spectrums for a trapped yeast cell and a PMMA particle considering a 60 s portion of the back-scattered signal acquired using lenses type 1A and 1B are provided in Figure 17A,B, respectively. As expected, the amount of light scattered by the yeast cell is higher than in the case of PMMA particle, despite of its smaller size (please see Figure 17A), thus contributing for a higher energy to be collected back by the photodetector. This is probably due to the higher refractive index of the yeast cell in comparison with PMMA [7,16,33]. Considering the *p*-values of the parwise statistical comparison between PMMA microspheres and yeast cells using the LDA single feature (please see the graphic of Figure 18), all the lenses allowed a statistical significant distinction between the two classes of particles (for a significance level of 0.05; p<0.05). Nevertheless, lenses type A are shown to perform comparatively better, concerning the differentiation ability. In fact, lens 1B was not characterized by a narrow power spectrum frequency peak amplitude for each particle type, on contrary of lenses type A (Figure 17B).

Indeed, for photopolymerization laser values of 5 and 10 μW, the difference between the final LDA-derived feature was more significant in statistical terms (e.g., leading to smaller *p*-values for the corresponding statistical comparisons) for lenses type A (1A and 2A) than type B (1B and 2BB, respectively)—Figure 18. However, the inverse is observed between lenses 3A and 3BB.

**Figure 19:**
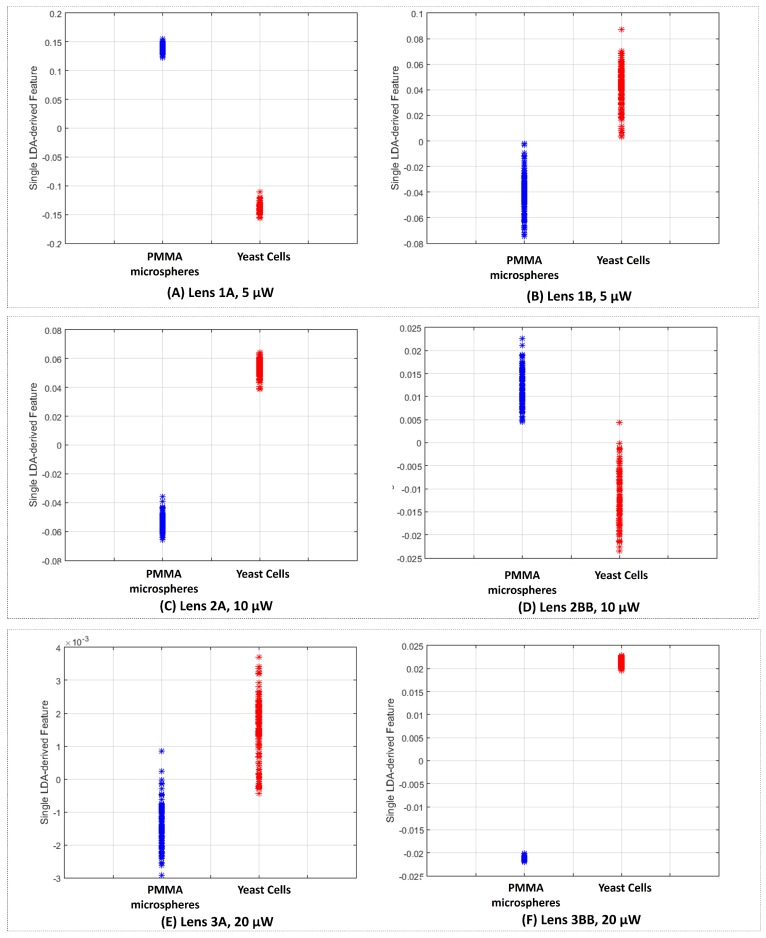
Comparison between LDA-derived feature values for 2-s short-term back-scattered signals corresponding to the five PMMA microspheres and five yeast cells for lenses (**A**) 1A, p=1.68E−11); (**B**) 1B (p=4.03E−05); (**C**) 2A (p=5.47E−08); (**D**) 2BB (p=3.27E−05); (**E**) 3A (p=2.30E−03) and (**F**) 3BB (p=4.86E−11).

By plotting the LDA-derived feature corresponding to all the 2-s back-scattered portions collected for all the PMMA particles and yeast cells analyzed (Figure 19), we can conclude that, overall, lenses type A allowed a better separability degree than lenses type B, except for the case with 20 μW photopolymerization laser power. Some hints can therefore be taken from the obtained results. Lenses type B (wider base, longer lengths) seem to be more suitable for optical trapping, because the resulting optical forces are higher in magnitude than in type A lenses (thinner or shorter). On the other hand, in average, lenses type A revealed to be more suitable for particles type differentiation using the back-scattered signal (considering photopolymerization laser power values of 5 or 10 μW). The contrary was observed for fabrication laser power of 20 μW. One possible reason for this behaviour of type A tips comes from the fact that its diameter approximately matches the diameter of the particle and, therefore, the underlying information of the back-scattered signal is provided only from the trapped particle. In the case of lenses type B, due to their larger diameter, the back-scattered signal is collected both from the trapped microparticle, but also from its surrounding environment, creating more noisy segments. Regarding photopolymerization laser power, 10 μW showed to be the option with more reproducible results. In fact, this photopolymerization laser value is the most suitable for fabricating micro-lenses with a good optical trapping performance yet allowing a good particles type differentiation ability. Nevertheless, we can see some variability between the performance of the different types of lenses obtained. Two major factors affecting its performance are the tip curvature radius (which depends on its length and the polymerization power), and the length (which influences both the thickness and the transversal extent of the field reaching the focusing tip). On top of these parameters, we have also to take into account that perturbations can arise during lenses fabrication, modifying the modal field distribution, especially for longer MM sections where the mode expansion is larger. Some reflection from the core/cladding boundary can also arise during the process. All these interdependencies lead to some degree of variability on the trapping and scattering collecting performance of the different types of lenses.

Overall it was observed that the lenses with longer MM length, and higher laser power, produced the least reproducible results. While it was possible to obtain lenses with such parameters having a very good performance, such as the case of tip 2B, it was hard to reproduce such results. This variability comes from the fact that long MM lengths create a larger mode expansion and more instability in the output field pattern, which then translates in a less reproducible polymerization pattern. Strategies for more stable modal excitation such as mode filtering, or polymerization at a wavelength where the fiber is SM, will surely allow to overcome these difficulties enabling the reproducible fabrication of more robust optical tweezers.

## 4. Conclusions

In this study, we propose a novel fabrication method of polymeric lensed fiber tips for simultaneous trapping and differentiation of trapped particles with improved trapping performance in comparison with its original version proposed in our previous studies. This variant fabrication method consists in the introduction of a MM section on the SM fiber and the assembly of a micro-lens on the MM section top due to a self-guided photopolymerization method. The influence of two types of factors on the final lenses geometry, trapping performance and trapped particle identification ability was also tested: the micro-lenses final base diameter, which depended on the MM section length; and the laser power value used in the photopolymerization process (influencing thickness and tip curvature radius). By introducing MM sections with different lengths, it was possible to obtain different types of microlenses which could be grouped into 2 types: Type A (lenses produced using shorter MM sections, with base diameters lower than 15 μm) and Type B (lenses produced using longer MM sections, with base diameters higher than 15 μm). We concluded that, by varying these fabrication parameters, we could obtain different micro-lenses adequate for different purposes. If we intend to obtain a lens uniquely to allow a strong and stable trapping of the selected target, the most suitable fabrication conditions include a longer MM section and a photopolymerization laser power of 10 μW, since micro-lenses type B obtained using this laser power exerted the maximal trapping forces on yeast cells from all the other lenses types. If the simultaneous trapping and particles type classification is intended, a compromise solution have to be found, since lenses Type A are more suitable for particles distinction through the back-scattered signal. We therefore concluded that the best option in this case would be a lens type A, fabricated using a photopolymerization laser power of 10 μW, since from the fabricated lenses Type A, the ones that exerted higher trapping forces were the ones fabricated considering that power value. However, all the micro-lenses produced with this novel method were able to exert higher force magnitude values on cells and synthetic targets, in comparison with the conventional fabrication method which only included the SM fiber. Lenses type A ensure an optical trapping two times stronger and lenses type B ten times stronger than SM fiber tweezers. Some variability affecting reproducibility was observed, particularly when longer MM length and higher powers were used. This arises from the fact that this type of structure yield a more expanded mode diameter, which can also be affected by reflections in the tip and core/cladding boundary, which is therefore more susceptible to fluctuations. Several strategies enabling a more stable pattern of the excitation radiation such as mode filtering and polymerization with a longer wavelength will surely improve on the reproducibility and quality of the produced lenses. In spit of all, the overall results are very encouraging as a big performance improvement was obtained when comparing any of the lens fabricated with the standard method using simple SM tips.

## Figures and Tables

**Table 1:** Polymeric fiber probes selected for detailed characterization in this study. Features analyzed include beam propagation profile (theoretical simulations), trapping forces and/or microparticles type differentiation ability using back-scattered signal.

Photopolymerization	Micro-Lens ID	Analysis Performed
Laser Power		
5 μW	1A	Trapping Forces Calculation, Back-scattered Signal-based
		differentiation ability, Theoretical Simulations
	1B	Trapping Forces Calculation, Back-scattered Signal-based
		differentiation ability, Theoretical Simulations
10 μW	2A	Trapping Forces Calculation, Back-scattered Signal-based
		differentiation ability, Theoretical Simulations
	2B	Trapping Forces Calculation, Theoretical Simulations
	2BB	Back-scattered Signal-based differentiation ability
20 μW	3A	Trapping Forces Calculation, Back-scattered Signal-based
		differentiation ability, Theoretical Simulations
	3B	Trapping Forces Calculation, Theoretical Simulations
	3BB	Back-scattered Signal-based differentiation ability

**Table 2:** Table summarizing the back-scattered signal parameters that contributed to LDA.

Type	Group	Number	Feature/Parameter
**Time Domain**	**Time Domain Statistics**	1	Mean (M)
		2	Standard Deviation (SD)
		3	Skewness (Skew)
		4	Kurtosis (Kurt)
		5	Interquartile Range (IQR)
		6	Entropy (E)
	**Time Domain Histogram**	7	$\mu_{Nakagami}$
**Frequency Domain**	**Discrete Cosine Transform (DCT)**	8 … 27	1st … 20th Coefficient ($E_{DCT}\left[l^{1}\right]$ … $E_{DCT}\left[l^{20}\right]$)
		28	Number of coefficients that capture 98% of the original signal ($N_{DCT}$)
		29	Total spectrum Area Under Curve (AUC) ($AUC_{DCT}$)
		30	Maximum peak amplitude ($Peak_{DCT}$)
		31	Total spectral power ($P_{DCT}$)
	**Wavelet Packet Decomposition**	32 … 37	Haar Relative Power 1st … 6th level ($E_{Haar}^{1}$ … $E_{Haar}^{6}$)
		38 … 43	Db10 Relative Power 1st … 6th level ($E_{Db10}^{1}$ … $E_{Db10}^{6}$)

**Table 3:** Simulation parameters values considered. RI—Refractive Index.

Simulation Parameters			
**Optical System**	Computational Grid	Dimensions (length × width)	500 μm × 70 μm
		Spatial resolution (length × width)	45.002 nm × 45.016 nm
	Waveguide	Polymeric Micro-lens Geometry	see table of Figure 4
		Polymeric Micro-lens RI	1.5200
		Single Mode Cladding RI	1.4510
		Single Mode Core RI	1.4575
		Multi-mode Mode Section Cladding RI	1.4510
		Multi-mode Mode Section Core RI	1.4590
	Optical Source	Wavelength	980 nm
		Duration	Continuous
	Media (distilled water)	RI	1.3270

**Table 4:** Fit parameters of experimental values.

	Theoretical	5 μW	10 μW	20 μW
slope	0.165	0.134	0.150	0.151
$d_{s}$, for L=0 (μm)	5.800	2.892	3.056	5.701

**Figure A1:**
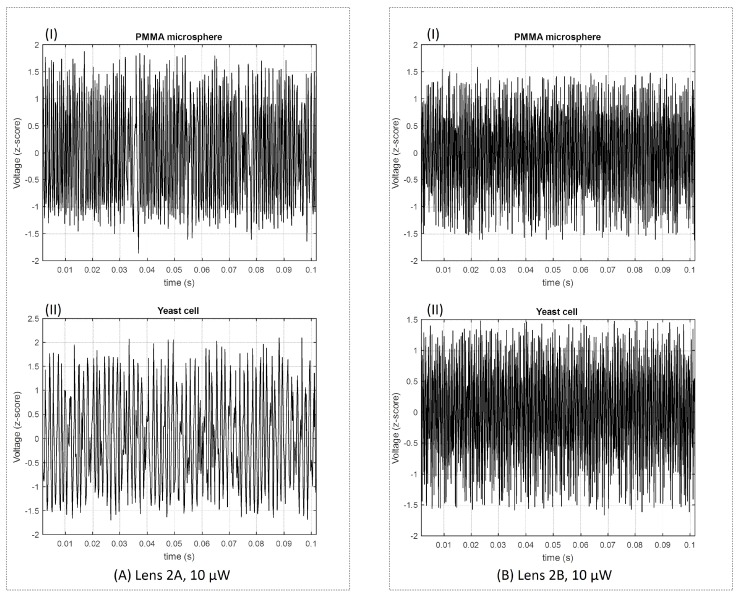
Sketches of the back-scattered signal acquired for (I) PMMA microsphere and (II) yeast cell for (**A**) micro-lens 2A and (**B**) 2BB (fabricated using a polymerization laser power of 10 μm).

**Figure A2:**
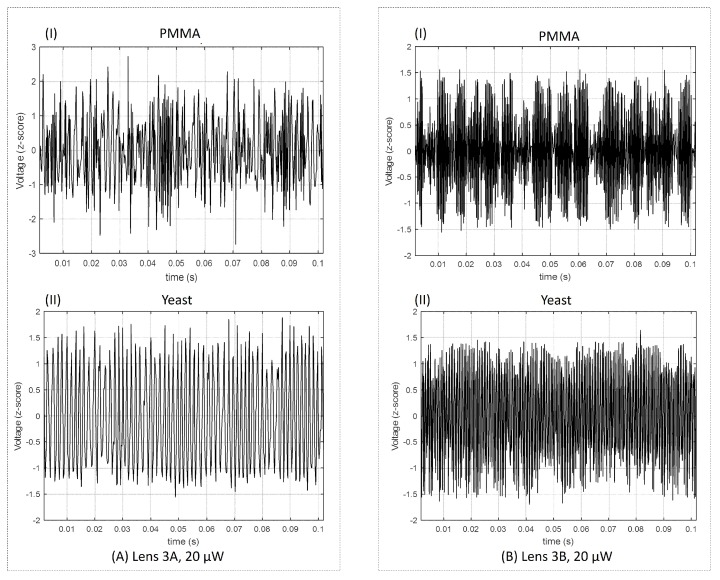
Sketches of the back-scattered signal acquired for (I) PMMA microsphere and (II) yeast cell for (**A**) micro-lens 3A and (**B**) 3BB (fabricated using a polymerization laser power of 20 μm).

## Abbreviations

The following abbreviations are used in this manuscript:

AUC	Area Under the Curve
DAQ	Data Acquisition Board
DCT	Discrete Cosine Transform
FDTD	Finite Differences Time Domain
FFT	Fast Fourier Transform
FIB	Focused Ion Beam
LDA	Linear Discriminant Analysis
MEEP	MIT Electromagnetic Equation Propagation
MM	Multi Mode
OT	Optical Tweezers
PDF	Probability Density Function
PMMA	Poly(methyl methacrylate)
RI	Refractive Index
SM	Single Mode
SNR	Signal-to-noise Ratio
